# Assessing the Living Well in the Community Program for people with disabilities in the context of COVID-19

**DOI:** 10.3389/fresc.2026.1671335

**Published:** 2026-02-13

**Authors:** Luke Santore, Catherine Ipsen, Tannis Hargrove, Hannah Pepprock

**Affiliations:** The Rural Institute for Inclusive Communities at The University of Montana, Missoula, MT, United States

**Keywords:** COVID-19, digitization, disability, independent living, online, rural, training

## Abstract

**Purpose:**

Living Well in the Community (LWC) is a health promotion intervention developed by RTC:Rural to support people with disabilities in building self-management of health, well-being, and community engagement skills. In response to the COVID-19 pandemic and the need for remote access, LWC was adapted for online delivery. This study evaluates outcomes from the program's online implementation between 2020 and 2023.

**Methods:**

The online version of LWC was delivered over ten weekly sessions via Zoom-based meetings and a dedicated website. The study examined outcomes from participants (*n* = 119) at 15 Centers for Independent Living (CILs) across the US. Participants completed pre- and post-program surveys assessing behavioral health (PROMIS-29), community engagement, healthcare utilization, and comfort with online communication. A total of 69 participants completed the post-surveys.

**Results:**

PROMIS scores showed insignificant improvements in behavioral health. However, participants reported a significant increase in hours spent on education, employment, or volunteering (from 7.42 to 11.13 h, *p* = .002). Self-reported confidence in managing chronic conditions remained high, with 89.5% of respondents rating themselves at 5 or lower on a 10-point scale (lower = more confident).

**Conclusion:**

While online delivery of LWC demonstrated reduced impact compared to prior in-person implementations, the program supported participants in maintaining stable health and engagement during a period of significant global disruption. While results were generally insignificant, they still suggest that online delivery of the LWC program may have been a valuable intervention for participants. Future research should investigate hybrid models and use larger sample sizes to assess program efficacy.

## Introduction

Approximately 41.9 million people in the United States, or 12.9% of the population, have a disability (1). Many people with disabilities develop secondary health conditions as a direct or indirect result of their primary disability. Some examples of secondary conditions include depression, hypertension, chronic pain, fatigue, and social isolation (2). Context-specific management of primary disabilities can often prevent or ameliorate secondary conditions (3). However, people with disabilities face systematic barriers that impede care. For example, people with disabilities experience lower quality and poorer access to housing, education, employment, transportation, and healthcare (4).

Systemic inequality is not a new experience for people with disabilities. The Independent Living (IL) philosophy emerged in the 1960's in response to historic institutionalization of people with disabilities, who often had decisions made for them, without them (5). The IL philosophy promotes agency and advocacy to maximize the independence, empowerment, and full inclusion of people with disabilities in society. Subsequently, IL skills include skills that enhance a disabled person's autonomy, control, or self-actualization, from tasks of daily living to personal care, use of accessibility equipment, employment skills, or social and advocacy skills.

Even with the presence of a comprehensive support system, independent living skills range from helpful to essential for people with disabilities. Persistent structural barriers to supports and services also make self-management skills crucial for people with disabilities to meet basic needs, manage health, and receive adequate care (6). In lieu of more ubiquitous and accessible disability social services, there is a clear need for self-management skills training that supports individual engagement in health behaviors. Strengthening these competencies would help prevent or reduce the prevalence of secondary conditions and improve overall well-being for people with disabilities. To this end, researchers at RTC:Rural developed and implemented the Living Well in the Community program (7).

### Living well in the community background information

The Living Well in the Community program (LWC - formerly known as Living Well with a Disability) was designed to build health behavior skills, confidence, and motivation to improve management of primary and secondary disabilities (7). Over 10 weekly sessions, LWC participants with disabilities first set quality of life goals, then learn and apply health promotion strategies to achieve these goals. LWC weekly content covers goal setting, building supports, healthy reactions, managing emotions, healthy communication, seeking information, eating well, physical activity, advocacy, and maintenance. The program was first evaluated in 1998–99 in a randomized trial with 246 consumers from Centers for Independent Living (CILs) across nine different states. Results showed that the LWC program reduced the incidence of secondary conditions, reduced health care utilization, and improved health-related quality of life (6, 8).

Development of LWC was based on two conceptual models including Antonovsky's Sense of Coherence (SOC) Model and Seligman's Attribution Style Model (7). Antonovsky described SOC in terms of (1) *comprehensibility*, the feeling that life is understandable, (2) *manageability*, the sense that one can influence life events, and (3) *meaningfulness*, the belief that barriers in life are worth overcoming. Seligman's Attribution Style Model posits that people fall along a continuum of attributes regarding life events. People who tend to give up or get overwhelmed by barriers describe situations as permanent, global, and related to personal factors. Conversely, people who are persistent tend to see barriers to solvable, specific, and not personal, which enables them to see each setback as a single event to overcome. Building on these models, the LWC program is grounded in helping people to identify meaningful goals in their lives and then to build tools and strategies to overcome barriers and move towards a more fulfilled state of being.

LWC was originally designed to be delivered in-person by CIL staff with an accompanying participant workbook. Due to workbook production costs along with travel barriers for some rural consumers, researchers at RTC:Rural began to explore alternate ways to offer the program. With funding from the National Institute on Disability, Independent Living, and Rehabilitation Research (NIDILRR), researchers developed an online LWC delivery website using a Community-Based Participatory Research (CBPR) approach called Iterative Participatory Curriculum Development (9). The resulting website mirrored the original LWC content but introduced additional modalities (e.g., videos, slideshows, and external links) for exploring concepts. More information on the LWC program can be found at https://healthycommunityliving.com/hcl/lwc-session/home/.

The online LWC curriculum was designed to be delivered either in-person or via hybrid delivery by CIL facilitators. However, due to the COVID-19 pandemic, the program transitioned to fully remote delivery utilizing the LWC website and a Zoom meeting format. This study aims to ascertain if the evidence-based LWC program remained effective when adapted to a remote delivery format, in the context of COVID-19.

### COVID impacts and contraindicators

The first fully remote LWC implementation started in mid-May 2020, approximately two months after the World Health Organization declared COVID-19 a pandemic. According to Centers for Disease Control and Prevention data, “rates of anxiety and depression among US adults were about four times higher between April 2020 and August 2021 than they were in 2019” (10) and global prevalence of anxiety and depression increased by 25% in the first year of the pandemic (11). Although less widely studied, evidence suggests that the mental health toll of COVID-19 on people with disabilities was dramatic, particularly for those with preexisting mental health conditions (12, 13). For example, in one survey (*n* = 127) 90% of people with disabilities reported “negative impacts on my mental health” (14) while in another (*n* = 1027), 38%–39% of respondents reported increased anxiety and stress and 18% reported more frequent feelings of despair (15). Compared to the general population, COVID-19 appeared to result in substantially worse mental health outcomes for people with disabilities.

Concurrent with declining physical and emotional health, barriers to services intensified, where people with disabilities reported losing access to home health providers (13, 16), occupational therapy, physical therapy, mental health services, transportation, and peer supports (17). Barriers to care often resulted in secondary health issues such as loss of muscle, increased pain, and declining function (17).

Although access to services declined, the emergence of tele-services filled some gaps and were recognized as beneficial for overcoming transportation and other community access barriers. Many providers and consumers, however, found tele-services inaccessible based on disability type (e.g., sensory, physical, or cognitive), technology costs, literacy issues, and inability to effectively utilize the new technology [e.g., slow internet speeds (18)].

In consideration of the mental health and access factors mentioned, implementation of the online LWC program during the COVID -19 pandemic may have impacted its efficacy, relative to pre-pandemic implementation.

## Methods

Evaluation of the LWC website and online format was conducted at 15 CIL study sites from 13 states between May 2020 and June 2023, in the midst of the COVID-19 global health crisis. CIL facilitators from identified sites participated in 10 h of asynchronous training on participant recruitment and LWC program delivery. The transition from in-person to remote delivery, however, required some additional training and delivery modifications. During study implementation, project staff held weekly meetings to support online facilitation skills development and to provide opportunities for peer support regarding unanticipated delivery challenges. To address participant computer access and literacy barriers, project staff purchased tablets with data plans for participant use and provided technical assistance to ensure that participants were able to sign into Zoom meetings, access the LWC website and communicate remotely.

The transition from in-person to online delivery also impacted participant recruitment. CIL facilitators were responsible for participant recruitment, which consisted of outreach to existing CIL consumers and through flyers and presentations at community partner events and locations. CIL facilitators shared that COVID-related health issues, participant reservations about using technology, and fewer opportunities for community outreach impacted these recruitment efforts. As a result, recruitment targets fell well below targeted projections.

### Participants

A total of 119 participants were recruited and responded to the pre-surveys. Of these, 69 (58.3%) responded to post-surveys administered at the conclusion of the LWC program. Participants were adults with disabilities recruited from CILs of different sizes in different geographic contexts. Study participants only needed to be CIL consumers. All participants took part in an introductory session that described the LWC program. They then provided informed consent to participate in the LWC program and complete the pre- and post- surveys. We used chi-square tests to compare pre-workshop data between responders (*n* = 69) and non-responders (*n* = 50), the outcomes of which can be found in the results.

### Measures

The following measures assessed disability, and program outcomes in behavioral health, health care utilization, community engagement, and technology use and comfortability.

### Qualitative feedback from facilitators

In response to the transition to fully remote delivery, staff held weekly meetings with facilitators, which included questions regarding their experiences with program recruitment, retention, and delivery of the program. We examined notes from these meetings to provide simple context for the statistical results, but never formally qualitatively coded them, as they were outside the scope of this paper.

### Disability

To assess disability type, we used the standard six disability questions found in the American Community Survey (ACS). These questions assess hearing, vision, cognition, ambulatory, self-care, and independent living disabilities. As a standardized measure, the ACS-6 can be compared across studies. The ACS-6 questions use a functional disability framework by asking about difficult performing functional tasks as opposed to diagnosis. For instance, the question about cognition asks, “Because of a physical, mental, or emotional problem, do you have difficulty remembering, concentrating, or making decisions?” rather than, for example, “Do you have a cognitive disability?” This minimizes underreporting of disability by focusing on the impact of disabilities instead of inherently variable self-identification or diagnostic labels (19).

#### PROMIS-29 profile v2.1

The Patient-Reported Outcomes Measurement Information System (PROMIS) instruments measure physical, mental, and social health and contain a fixed number of items from seven domains (depression, anxiety, physical function, pain interference, fatigue, sleep disturbance, and ability to participate in social roles and activities). PROMIS instruments are designed to assess general health-related quality of life, rather than disease-specific measures. For this study, we utilized the PROMIS-29 Profile v2.1. The PROMIS-29 contains four questions in each of the seven domains as well as a pain intensity rating scale (20). Responses are measured on a 1–5 Likert-type scale, and domain scores range from 4 to 20 points. Total scores range from 28 to 140 points. Questions for which the scale moved in the opposite direction (Sleep 109 and Sleep 116) were re-coded so that the scale for all measures moved in the same direction, where higher scores indicate improved behavioral health.

#### Healthcare utilization

We measured healthcare utilization in terms of number of hospitalizations, emergency rooms visits, outpatient procedures, physician visits, medications, and pain medications over the last two months. When answering these six items, participants were inadvertently given the ability to enter text answers, instead of being restricted to numeric answers. Subsequently, many text answers required manual conversion to numeric values. All text numbers were converted to their numeric equivalents (i.e., “one” or “three times” to 1 or 3). All instances of “none,” “never,” or “n/a” were converted to 0. Entries of “N+,” “N?” or “N or more” were converted to N. (i.e., “10+,” “8?” and “6 or more” converted to 10, 8, and 6, respectively). Entries of “X-Y “or “X to Y” were converted to the average of the range (i.e., 4–6 and 7–8 converted to 5 and 7.5 respectively). A handful of unique entries were marked as missing as participant intent was unclear. Finally, participants who reported their number of medications as “a lot,” “too many to count,” and “all of them,” were converted to missing. It is unlikely that this survey error had a meaningful effect on the results, other than a smaller reported mean number of medications.

#### Community engagement questionnaire

Community engagement was measured by asking participants how many times they visited a location (i.e., grocery store, public park) or participated in an activity (i.e., socializing, religion) in the last seven days. The Community engagement Questionnaire contains a total of sixteen questions about community participation. Thirteen of the questions ask about the number of times participating in an activity in the past seven days (healthcare visits, trips to grocery or big box stores, etc.). The last three questions ask about the number of hours spent participating in an activity in the last seven days (education, employment, and volunteering). We've used this measure in past studies to measure longitudinal changes in community participation (21).

#### Technology use and comfortability

After the LWC program moved online, we decided to ask participants about their internet use. We sourced indices from Ledbetter's (22) Online Communications Scale. This scale is comprised of 36 items split into six indices. They are categorized into comfort using technology (*n* = 6, i.e., “I feel comfortable using email), self-disclosure (*n* = 7, i.e., It is easier to disclose personal information online), apprehension (*n* = 8, i.e., I feel awkward when communicating online), miscommunication (*n* = 5, i.e., Miscommunicating occurs frequently online), social connection (*n* = 6, i.e., Losing internet access would not change my social life at all), and ease of use (*n* = 4, i.e., Online communication is convenient). This measure has been used in previous studies to assess frequency, comfortability, and attitude towards online communication (23).

#### Condition management

Post-test participants were asked to rate their confidence on a 1–10 scale (where 1 = totally confident and 10 = not at all confident) to the question “After taking this workshop I am more confident that I can manage my chronic conditions.**”**

## Results

Context for LWC Implementation: During weekly meetings, CIL facilitators reported that both recruitment and retention were significant issues due to online-only delivery and COVID-19 impacts. CIL facilitators also expressed frustrations related to contacting people and onboarding them to the online format. They shared that many rural CIL consumers lacked the experience and technology equipment to participate, and highlighted difficulties managing the online group format. Additionally, they shared that stay at home and social distancing protocols undermined many of the foundations of the LWC curriculum, such as community participation and social supports. These qualitative findings highlight barriers to both recruitment and implementation. Despite these barriers, the participants who successfully engaged with the LWC program were diverse in age, gender, education, and disability type, although less so in race.

Table 1 details participant characteristics (*n* = 119). Ages ranged from 18 to 84 years with a mean age of 44.55. Participants were predominantly White (69.7%), non-Hispanic (92.2%), and female (66.1%). The majority of participants were not employed (72.6%) and did not participate in volunteer activities (54.3%). 82.8% of participants reported an annual income of $20,000 or less. We collapsed “Native Hawaiian or Pacific Islander,” (*n* = 0) “Asian,” (*n* = 1) and “Other” (*n* = 4) into the Other category due to low subsample sizes. Disability types and race add up to greater than 100% because participants were able to select more than one disability and race, respectively.

**Table 1:** Participant characteristics.

Demographic Category	*N*	Percent
Age		
Range	18–84	
Mean age	44.55	
18 to 29^a^	25	21.6%
30 to 44	33	28.4%
45 to 64^a^	47	40.5%
65+	11	9.5%
Gender		
Female	78	66.1%
Male	37	31.4%
Other	3	2.5%
Race		
White^a^	83	69.7%
Black/African American	23	19.3%
American Indian/Alaska Native	5	4.2%
Other	5	4.2%
Relationship Status		
Married	14	12.0%
Never been married	52	44.4%
Member of an unmarried couple	9	7.7%
Separated/Divorced/Widowed	42	35.9%
Education		
Grades 9 through 11 (some high school)	7	5.9%
Grade 12 or GED (high school graduate)	40	33.9%
Some college or technical school training	38	23.7%
Associate or technical degree	15	12.7%
Bachelor's degree	20	16.9%
Master's degree or higher	8	6.8%
Disability Type		Percent of Cases
Hearing	9	7.6%
Vision	27	23.1%
Cognition	54	46.6%
Ambulatory	60	50.8%
Self-Care	33	28.2%
Independent Living^a^	58	50.4%

a Statistically significant Chi-Square test differences between participants who completed both the pre- and post-test and participants who only completed the pre-surveys.

While the pre- and post-test groups shared similar demographic characteristics, a significantly larger proportion of White people (69.7% vs. 76.8%, *p* = .039) and a significantly smaller proportion of people with independent living disabilities (50.4% vs. 40.6%, *p* = .008) completed the post-test. When age was organized into categories, a significantly smaller proportion of 18- to 29-year-olds (21.6% vs. 14.7%, *p* = .029) and a significantly larger proportion of 45–64 year olds (40.5% vs. 48.5%, *p* = .028) completed the post surveys.

To determine if any systematic differences may have impacted the results, we used chi-square tests to compare pre-workshop data between those who completed both the pre- and post-workshop surveys (*n* = 69) with those you did not (*n* = 50). While the pre- and post-test groups shared similar demographic characteristics, a significantly larger proportion of White people (69.7% vs. 76.8%, *p* = .039) and a significantly smaller proportion of people with independent living disabilities (50.4% vs. 40.6%, *p* = .008) completed the post-test. When age was organized into categories, a significantly smaller proportion of 18- to 29-year-olds (21.6% vs. 14.7%, *p* = .029) and a significantly larger proportion of 45–64 year olds (40.5% vs. 48.5%, *p* = .028) completed the post surveys. After examining these demographic differences, we looked at differences in health outcomes before and after the LWC program.

Within this context, Table 2 reports paired samples t-tests between pre- and post- data on key outcome variables, as well as a post-test-only question that asks participants if the workshop improved their confidence managing their chronic condition(s). For all of the seven PROMIS-29 indices, lower scores indicate better behavioral health outcomes. PROMIS-29 indices, as well as the cumulative index, exhibited marginal, insignificant changes in means between the pre- and post-workshop surveys.

**Table 2:** Pre- and post-test results for the online administration of the LWC health promotion intervention.

Measure	Mean Pre (SD)	Mean Post (SD)	Two-Tailed *p*
PROMIS-29 Cumulative Index (lower is better, range of 28–140)	73.57 (23.86)	71.94 (24.4)	0.42
PROMIS-29 Indices (lower is better, range of 4–20)			
Physical Function	11.49 (5.09)	11.95 (4.9)	0.10
Anxiety	9.32 (3.8)	9.02 (3.89)	0.36
Depression	8.73 (4.1)	8.23 (4.28)	0.21
Fatigue	10.71 (4.8)	10.76 (4.72)	0.91
Sleep Disturbance	12.03 (4.27)	11.45 (4.3)	0.19
Ability to participate in social roles and activities	9.82 (4.54)	9.73 (4.5)	0.85
Pain Interference	11.05 (5.1)	10.83 (5.42)	0.67
Healthcare Utilization (lower is better)			
Hospitalization	0.11 (0.44)	0.83 (3.57)	0.12
Emergency Room Visits	0.27 (0.78)	0.32 (0.8)	0.50
Outpatient Procedures	0.26 (0.7)	0.21 (0.631)	0.65
Physician Visits	2.16 (2.66)	1.93 (2.09)	0.55
Medications	5.03 (4.83)	5.76 (5.24)	0.12
Pain Medications	0.84 1.2)	0.97 (1.35)	0.16
Community Engagement (higher is better)			
Number of visits in the past 7 days			
Grocery stores	1.97 (2.38)	1.77 (1.86)	0.41
Doctor or other healthcare provider	0.79 (1.33)	0.84 (1.33)	0.79
Pharmacies	0.47 (0.69)	0.56 (1.86)	0.58
Restaurants	1.15 (1.86)	0.92 (1.46)	0.33
Box stores	0.89 (1.61)	0.91 (1.4)	0.92
Public parks or recreation areas	0.83 (1.72)	0.72 (1.62)	0.63
Exercise facilities	0.45 (1.22)	0.47 (1.5)	0.95
Shopping malls	0.19 (0.69)	0.20 (0.76	0.87
Active recreation (sports, fishing, etc.)	1.27 (2.1)	1.45 (2.47)	0.55
Socializing outside the home	1.81 (2.37)	1.62 (1.82)	0.50
Religious activities	1.07 (1.96)	0.67 (1.49)	0.07
Community activities such as voting	0.52 (1.29)	0.53 (0.92)	0.93
Entertainment (movies, sporting events, etc.)	0.95 (2.2)	0.73 (1.95)	0.49
Number of hours in past 7 days (higher is better)			
Employment	4.05 (10.43)	5.00 (10.55)	0.37
School or education	1.05 (3.02)	2.98 (8.37)	**0**.**06**
Volunteering	2.26 (6.32)	3.06 (7.59)	0.44
Aggregated Community Engagement (higher is better)			
Number of visits in the past 7 days	12.42 (12.79)	10.80 (9.79)	0.14
Number of hours in the past 7 days	7.42 (14.87)	11.13 (15.74)	**0**.**02**
Condition Management			
After taking this workshop, I am more confident that I can manage my chronic conditions.	**N**	**Percent**	
1- Totally confident	21	31.8%	
2	9	13.6%	
3	7	10.6%	
4	10	15.2%	
5	3	4.5%	
6	9	13.6%	
7	3	4.5%	
8	0	0.0%	
9	0	0.0%	
10-Not at all confident	4	6.1%	

Bold values indicate significant findings.

As for healthcare, fewer healthcare interactions and medications are generally perceived as desirable, as this might indicate the LWC program successfully increased participants' self-management of primary and secondary conditions. Mean number of medications prescribed by a doctor taken over the last two months had an insignificant increase (5.03–5.76 *p* = .12) while mean physician visits had an insignificant decrease (2.16–1.93 *p* = 0.55). Emergency room visits, outpatient procedures, and pain medications had insignificant variations in means. Mean nights hospitalized in the last two months had an insignificant increase (0.11–0.83 *p* = .12). The size of this increase can be attributed to the small number of participants who experienced any nights of hospitalization (pre-test *n* = 10, post-test *n* = 8) allowing individuals to greatly skew the sample. The variance in the pre-test was relatively low (mean of 0.11, variation of 1.022) while the variance in the post-test was high due to two participants each reporting 20 nights in hospital (mean of 0.83 variance 12.34).

All changes in Community engagement means were insignificant, with participation in religious activities approaching significance (1.07–0.67, *p* = .07). However, when all of these items were aggregated, the mean instances of community participation show a decrease that approaches significance (12.42–10.8, *p* = 0.14).

For employment, school, and volunteering, participants were asked how many hours they spent on those activities in the last seven days. Participants reported higher but insignificant increases in mean hours spent on employment or volunteering. Participants reported a significant increase in mean hours spent on school or education (1.05–2.98, *p* = .006). When aggregated, hours spent on any of these three activities increased significantly (7.42–11.13, *p* = .002). Finally, the majority of participants (89.5%) rated their confidence in condition management after completing the workshop at a five or lower and 55.2% of participants rated their confidence at a two or lower, indicating relatively strong levels of confidence in the post-test group.

Table 3 contains Ledbetter's (22) Online Communications Scale, which we added after COVID-19 forced the program online. The cumulative score simply combines all six indices.

**Table 3:** Cumulative indices.

Online Communications Scale Dimensions (lower is better)	Mean Pre (SD)	Mean Post (SD)	Two-Tailed *p*
Dimension 1 Tech comfortability	2.71 (1.73)	2.68 (1.85)	.915
Dimension 2 Self-Disclosure	4.58 (1.56)	4.48 (1.53)	.616
Dimension 3 Apprehension	4.05 (1.24)	3.96 (1.3)	.632
Dimension 4 Miscommunication	4.31 (1.37)	4.47 (1.48)	.445
Dimension 5 Social Connection	3.68 (1.79)	3.55 (1.62)	.551
Dimension 6 Ease of use	3.06 (1.66)	2.97 (1.5)	.638
Cumulative Score	3.71	3.67	.675

Given the large number of statistical comparisons conducted across multiple outcome measures, we acknowledge an increased risk of type I error. To mitigate this, we applied a Bonferroni correction to significant *p*-values. While this is conservative and may increase the risk of type II error, it helps support the robustness of our conclusions.

## Discussion

The COVID-19 pandemic disrupted healthcare systems, social support networks, and daily routines across the world. We know that rates of depression and anxiety quadrupled in the general US population, and also dramatically increased for people with disabilities (15, 24). Historically, in-person administration of the LWC program showed statistically significant improvements across many of these domains (6, 8). In contrast, our findings were less substantial and largely insignificant, suggesting that online delivery may have been less effective. However, these results must be interpreted within the extraordinary context of COVID-19. The absence of substantial or significant deterioration across all of our measures may be evidence of a successful intervention that helped participants maintain relative stability.

Building on this, we anticipated that online participation requirements might enhance consumers' confidence in accessing other online networks and resources during the pandemic. However, none of the six dimensions in the Online Communications Scale presented substantial or significant results. In fact, of the 36 items in these scales, the only significant difference was email, where participants reported a significant and substantial increase in confidence. Email was the primary method of group communication, access to weekly Zoom meetings, and survey completion. Beyond this isolated result, the online administration of the LWC program did not appear to affect comfortability with technology use and online communication, although future research would be needed to clarify this finding.

As for healthcare utilization, we expected the COVID-19 pandemic to increase healthcare utilization due to illness, except for routine physician visits. Contrary to our expectations, there were no significant findings. Other research found that people with disabilities had higher rates of delayed or forgone healthcare utilization at the height of the pandemic, likely due to the acute risk of contracting COVID-19 (25, 26). Although our lack of significant findings may point towards a stabilizing effect for LWC participants, it remains difficult to determine the program's precise role in these patterns.

Restricted access to healthcare and community resources during the pandemic amplified the importance of self-management skills. The LWC programs' emphasis on health and community related self-management and independent living skills may have bolstered self-reliance when it was greatly needed. For example, participants reported significantly more hours spent on education, volunteering, or employment (7.42 to 11.13), an unexpected increase through the pandemic. While most pre- and post-tests were insignificant, scores remained generally consistent, potentially indicating some level of protection against poor behavioral health outcomes associated with COVID-19. It is also important to note that for the small sample size, substantial variability in pre- post-test scores would be needed to produce significant differences.

Finally, the post-test measure of confidence provides additional insight. The efficacy of online administration of the LWC program was supported by the post-test measure of confidence, where the vast majority of participants agreed with the statement, “After taking this workshop, I am more confident that I can manage my chronic conditions.” While it is difficult to draw strong conclusions, this collectively suggests that participation in the LWC workshop may have acted as a protective factor against the mental health risks of the pandemic. Further research may discover online administration of the LWC can serve as an effective health and community engagement tool for people with disabilities.

### Limitations

This study has several limitations. First, the study lacked a control group with which to compare results over time. Particularly in the context of the COVID-19 pandemic, this severely limited what we were able to conclude about the program effectiveness. For example, a comparison group cohort may have shown declining behavioral health outcomes in contrast to the relative stability of LWC participants. Additionally, we collected data over a very brief time period. This study would have benefited from more than one post-test, particularly given the extraordinary context of COVID-19.

The small sample size is another limitation of this study. We had significant difficulties recruiting participants to the online program and were not successful in our follow-up data collection efforts. In part, this was related to a number of staffing changes mid-stream and difficulties related to online survey data collection (e.g., survey links going to junk mail). The end result was a total sample of 119 and a post-test sample of 69, which are on the lower end of acceptability for the analyses we conducted. An *a priori* power analysis for the study indicated a sample size closer to 160.

Finally, there were degrees of response bias in our sample, where those who did and did not provide follow-up data may have been systematically different from one another. We found some evidence of this when comparing post-test responders and non-responders.

## Conclusion

Despite the extraordinary disruptions posed by the COVID-19 pandemic, participants in the LWC program reported relatively stable health outcomes, modest gains in community participation, and high confidence in managing chronic conditions. While most changes in pre- and post-test measures were not statistically significant, these findings suggest that the online LWC program may have helped buffer against negative health impacts of the pandemic. As health promotion interventions increasingly move toward virtual delivery, further research is needed to evaluate how online formats can effectively support independent living skills and well-being for people with disabilities.

## Data Availability

The raw data supporting the conclusions of this article will be made available by the authors, without undue reservation.
